# Abdominal surgery induces long-lasting changes in expression and binding of CTCF with impact on Major Histocompatibility Complex II transcription in circulating human monocytes

**DOI:** 10.1371/journal.pone.0293347

**Published:** 2023-10-25

**Authors:** Benedikt Hermann Siegler, Jan Niklas Thon, Marc Altvater, Judith Schenz, Jan Larmann, Markus Alexander Weigand, Sebastian Weiterer

**Affiliations:** Medical Faculty Heidelberg, Department of Anesthesiology, Heidelberg University, Heidelberg, Baden-Württemberg, Germany; Charité Universitätsmedizin Berlin CVK: Charite Universitatsmedizin Berlin - Campus Virchow-Klinikum, GERMANY

## Abstract

**Background:**

Postoperative immunosuppression has been recognized as an important driver of surgery-related morbidity and mortality. It is characterized by lymphocyte depression and impaired monocyte capability to present foreign antigens to T-cells via Major Histocompatibility Complex, Class II (MHC-II) molecules. In patients with postoperative abdominal sepsis, we previously detected a persisting differential binding of the CCCTC-Binding Factor (CTCF), a superordinate regulator of transcription, inside the MHC-II region with specific impact on human leucocyte antigen (HLA) gene expression. In this prospective exploratory study, we investigated to which extent major surgery affects the MHC-II region of circulating CD14^+^-monocytes.

**Results:**

In non-immunocompromised patients undergoing elective major abdominal surgery, a postoperative loss of monocyte HLA-DR surface receptor density was accompanied by a decline in the transcription levels of the classical MHC-II genes *HLA-DRA*, *HLA-DRB1*, *HLA-DPA1* and *HLA-DPB1*. The surgical event decreased the expression of the transcriptional MHC-II regulators CIITA and CTCF and led to a lower CTCF enrichment at an intergenic sequence within the HLA-DR subregion. During the observation period, we found a slow and only incomplete restoration of monocyte HLA-DR surface receptor density as well as a partial recovery of *CIITA*, *HLA-DRA* and *HLA-DRB1* expression. In contrast, transcription of *HLA-DPA1*, *HLA-DPB1*, *CTCF* and binding of CTCF within the MHC-II remained altered.

**Conclusion:**

In circulating monocytes, major surgery does not globally affect MHC-II transcription but rather induces specific changes in the expression of selected HLA genes, followed by differential recovery patterns and accompanied by a prolonged reduction of CTCF expression and binding within the MHC-II region. Our results hint toward a long-lasting impact of a major surgical intervention on monocyte functionality, possibly mediated by epigenetic changes that endure the life span of the individual cell.

## Introduction

Major surgery and the associated trauma are considered to be an element in suppressing cellular immunity [1]. Post-operative immunosuppression not only impedes the process of healing, but also increases the risk of severe infectious complications, multi-organ dysfunction, sepsis and death [1–4]. The innate immune system, which is activated following traumatic tissue injury, seems to play a central role in perioperative immune-modulation [5,6]. With the capability to present foreign antigens to CD4^+^-T-lymphocytes via Major Histocompatibility Complex, Class II (MHC-II) molecules, monocytes represent the first line of defence by mediating the initial host immune response [7].

As an acknowledged marker of global immune-competence in systemic inflammatory reactions, monocyte surface expression of the human leucocyte antigen DR (HLA-DR) has been widely studied via flow cytometry during different types of surgery [8–12]. Surgical trauma commonly induces a drop of monocyte HLA-DR surface expression and the extent of this reduction correlates with the incidence of postoperative surgical and infectious complications [13,14]. On the cellular level, loss of HLA-DR surface expression has been associated with impaired key cellular immune functions including cytokine production and the process of antigen presentation [15,16].

The mechanisms required to present antigenic peptides on the cell surface are particularly regulated on the level of gene transcription [17]. Target genes are located in the ~700 kb spanning MHC-II region on the short arm of human chromosome 6 (6p21.3) [18]. This region contains genetic information for alpha- and beta-chain components of the classical HLA-isotypes (HLA-DP, HLA-DQ and HLA-DR), for proteins aiding the process of antigen presentation (HLA-DO and HLA-DM) as well as for molecules not involved in antigen presentation [18–20]. Within the promoter regions of all HLA-genes, conserved cis-acting X-Y-elements are bound by distinct DNA-binding (co)factors that in turn recruit and interact with the Class II Major Histocompatibility Complex Transactivator (CIITA). This interplay results in the formation of a multimeric enhanceosome complex and the initiation of the general transcription machinery [21]. In HLA expressing Raji-cells–a B-cell line–the enhanceosome complex was shown to interact with the CCCTC-Binding Factor (CTCF) at an intergenic X-box like region inside the MHC-II, thereby modulating the transcription of spatially related *HLA-DRB1* and *HLA-DQA1* genes [22,23].

CTCF is ubiquitously expressed in mammalian cells and has been recognized as a genome-wide regulator of gene transcription [24]. The multivalent functions of CTCF include the blockade of enhancers, the higher-order modulation of chromatin conformation as well as the insulation of transcriptionally active regions [24,25]. It recognizes approximately 60,000 to 80,000 mostly intergenic genomic target sites [26,27], ten of which have been identified within the human MHC-II region [28]. Recently, we investigated the role of CTCF-binding within the MHC-II and its relevance for HLA gene expression in circulating monocytes of patients with sepsis-induced immunosuppression. At the time of diagnosis, sepsis following abdominal surgery was associated with increased CTCF occupancy at intergenic sites adjacent to classical HLA genes, accompanied by reduced transcription of *HLA-DRA*, *HLA-DRB1*, *HLA-DPA1* and *HLA-DPB1* [29]. These observations indicate that CTCF modulates the transcriptional MHC-II response of functionally impaired antigen-presenting cells during critical illness.

In the present study, we analysed perioperative immune capacity including monocyte function in non-immunocompromised patients undergoing elective major abdominal surgery. We investigated to which extent major surgery affects the MHC-II region as well as its superordinate regulators CIITA and CTCF in circulating CD14^+^-monocytes.

## Results

### Patients’ characteristics

**Fig 1:**
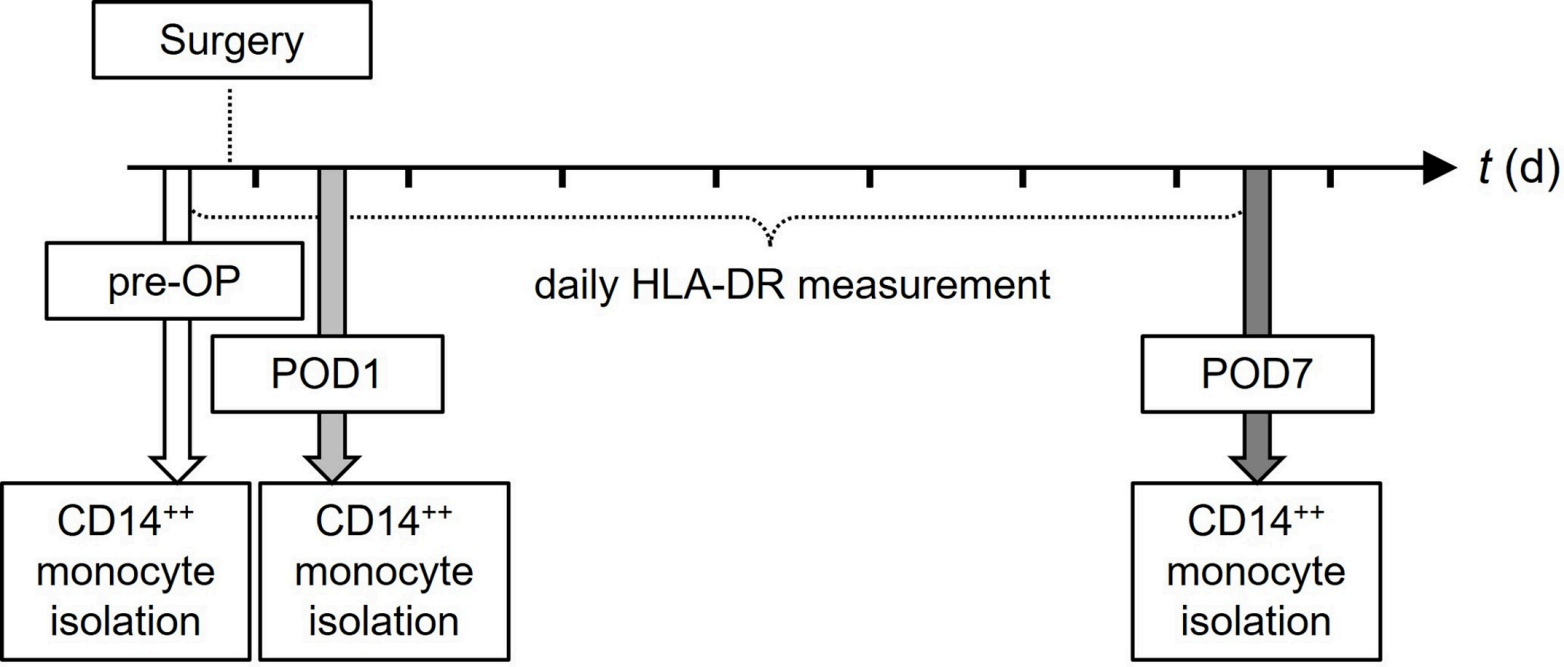
Study design. Ten patients undergoing major abdominal surgery were included. Blood samples for experimental workflow including isolation of CD14^+^-monocytes were taken before surgery (`pre-OP´) as well as on postoperative days one (`POD1´) and seven (`POD7´). Daily measurements of HLA-DR surface expression on CD14^+^-monocytes were performed until POD7.

Overall, 10 patients (five female and five male individuals) with a mean age of 68 years (range 51–79 years) participated in this study. All patients underwent elective major abdominal surgery including gastroesophageal, hepato-biliary and pancreatic operations. None of the patients suffered from autoimmune diseases, took immune-suppressive medication or showed any sign of infection on laboratory tests prior to study inclusion. Clinical and socio-demographic characteristics of the investigated study cohort are listed in **S1 Table**, while **Fig 1** visualizes the study design.

Blood samples were taken before surgery (`pre-OP´) as well as on the first and seventh postoperative day (`POD1´ and `POD7´). Global perioperative immune function was quantified through (i) evaluation of cytokine secretion capacity and *ex vivo* cellular responsiveness to inflammatory stimulation and (ii) daily measurement of antigen presentation capacity via flow cytometry (**Fig 1**).

### Surgery increases secretion of inflammatory cytokines

**Fig 2:**
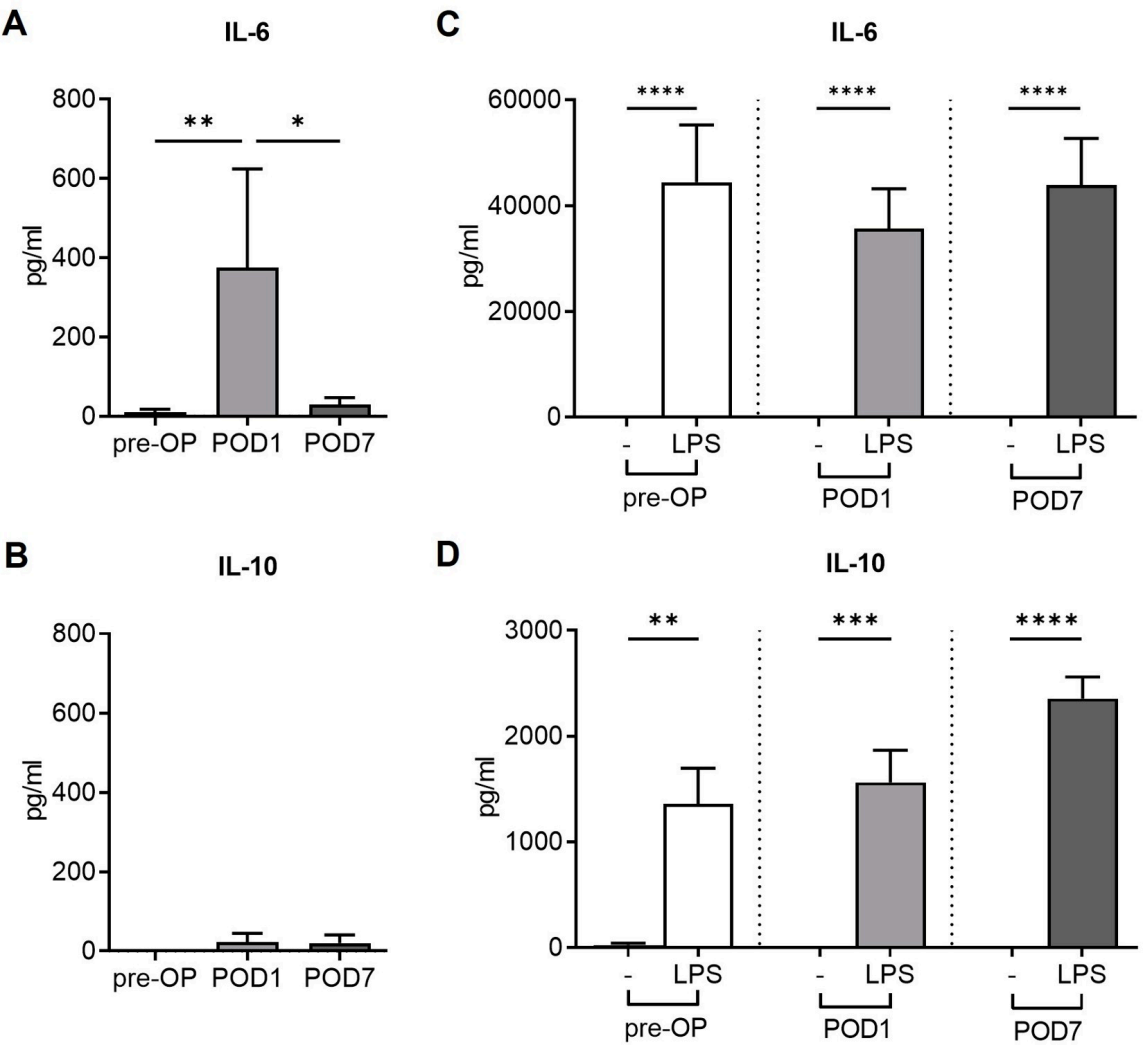
Secretion of inflammatory cytokines. Cytokine levels in plasma and supernatants from ex-vivo stimulated samples before surgery (`pre-OP´) as well as on postoperative days one (`POD1´) and seven (`POD7´). (A) IL-6 and (B) IL-10 levels in plasma (**p<0.01, *p<0.05, Mann-Whitney U test, n = 10 patients, mean + SEM). (C) IL-6 and (D) IL-10 levels in supernatants after ex-vivo stimulation with bacterial lipopolysaccharide (LPS; ****p<0.0001, ***p<0.001, **p<0.01, Mann-Whitney U test, n = 10 patients, mean + SEM).

Compared to pre-operative baseline levels (`pre-OP´), surgery led to an increase of pro-inflammatory IL-6 plasma concentrations on POD1 (mean 10 pg/ml vs. 375 pg/ml, **p<0.01, pre-OP vs POD1, **Fig 2A**). In contrast, no surgery-induced change was observed in anti-inflammatory IL-10 levels (mean 0.0 vs. 23 pg/ml, pre-OP vs POD1, **Fig 2B**). The *ex vivo* responsiveness to a secondary inflammatory stimulus was tested by incubating whole blood with the bacterial Toll-like receptor 4 agonist lipopolysaccharide (LPS). LPS stimulation strongly increased IL-6 and IL-10 levels before surgery as well as on POD1 (**Fig 2C and 2D**) with no difference observed in individual delta compared to unstimulated control (**S1 Fig**), indicating sustained perioperative reactivity of circulating immune cells in terms of cytokine secretion.

### Surgery induces a drop of monocyte surface HLA-DR expression and reduces classical HLA-DR and -DP gene expression

**Fig 3:**
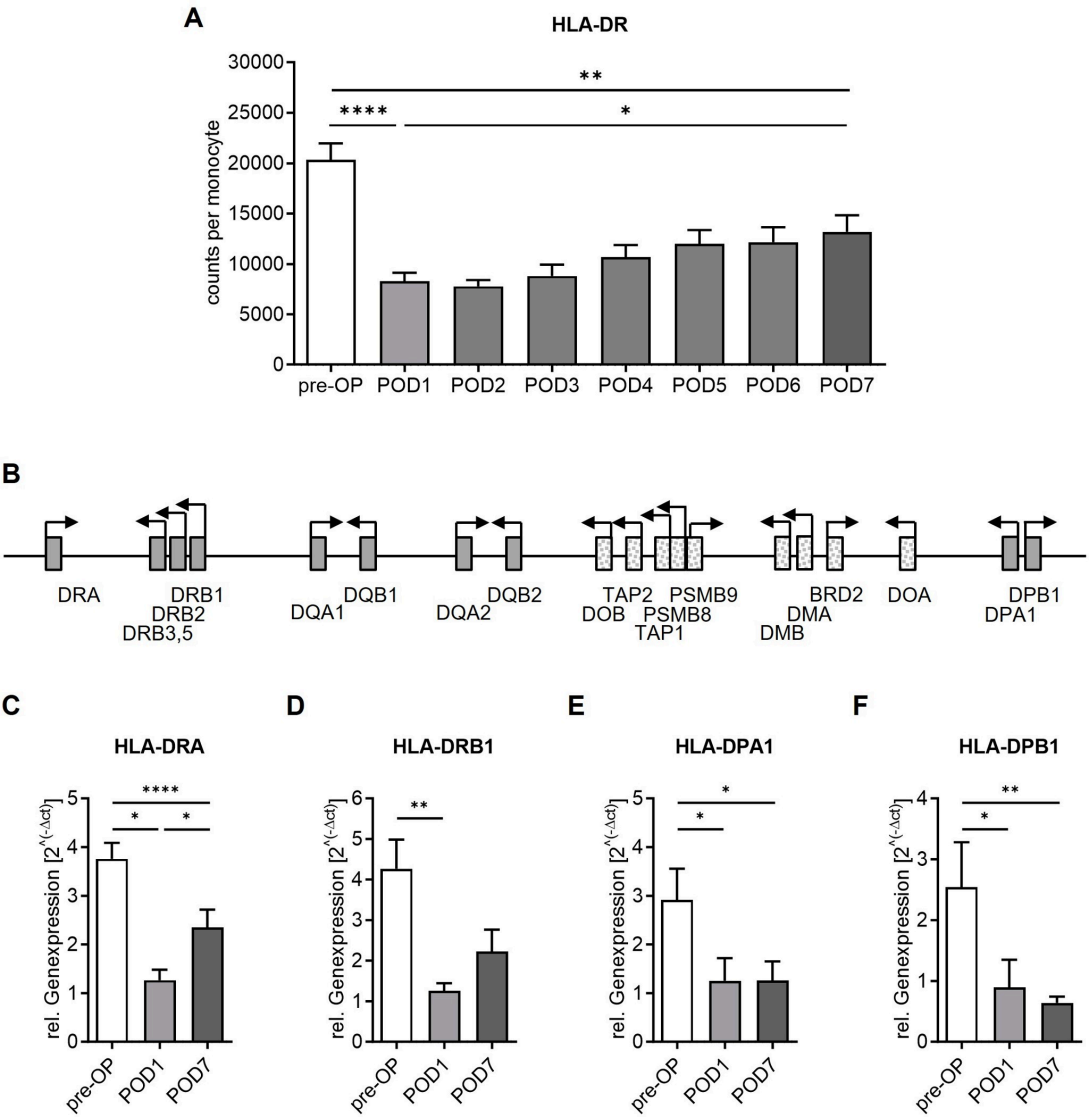
HLA-DR receptor density and expression of classical HLA genes. Samples were analyzed before surgery (`pre-OP´) as well as on postoperative days one (`POD1´) and seven (`POD7´). (A) Surface expression of HLA-DR on CD14^+^-monocytes (****p<0.0001, **p<0.01, *p<0.05, Mann-Whitney U test, n = 10 patients, mean + SEM). (B) Schematic diagram of human chromosome 6p21.3 showing the relative positions of HLA genes within the MHC-II region (classical HLA genes are shown as filled boxes). (C) to (F) RNA from isolated human CD14^+^-monocytes was used for reverse transcription and subsequent qPCR experiments using TaqMan Assay against classical HLA-DR and -DP subtypes (****p<0.0001, **p<0.01, *p<0.05, Mann-Whitney U test; n = 10 patients, mean + SEM).

Next, we used flow cytometry to analyse monocyte HLA-DR surface expression as surrogate of antigen presentation capacity via HLA class II surface molecules and global marker of immune competence. Surgery induced a strong drop of HLA-DR expression on CD14^+^-monocytes (mean 20,318 (pre-OP) vs. 8,297 (POD1) molecules per monocyte, ****p<0.0001, **Fig 3A**). Since antigen presentation is primarily controlled on the transcriptional level [17], further experiments focused on the expression of associated classical HLA genes within the MHC-II region (**Fig 3B**). RNA from circulating CD14^+^-monocytes was extracted and subsequent gene expression analysis was performed for classical HLA isotypes (*HLA-DP*, *HLA-DQ* and *HLA-DR*). At POD1, patients displayed a decline in mRNA expression of HLA-DR and HLA-DP subunits (*HLA-DRA*, *HLA-DRB1*, *HLA-DPA1* and *HLA-DPB1*, *p<0.05 and **p<0.01, pre-OP vs POD1, **Fig 3C**). No differences were observed in the transcription of *HLA-DRB3*, *HLA-DRB5* or the *HLA-DQ* subunits A1, B1, A2 and B2 (**S2 Fig**).

## Surgery reduces expression of CIITA and CTCF and selectively decreases intergenic CTCF occupancy within the MHC-II region

**Fig 4:**
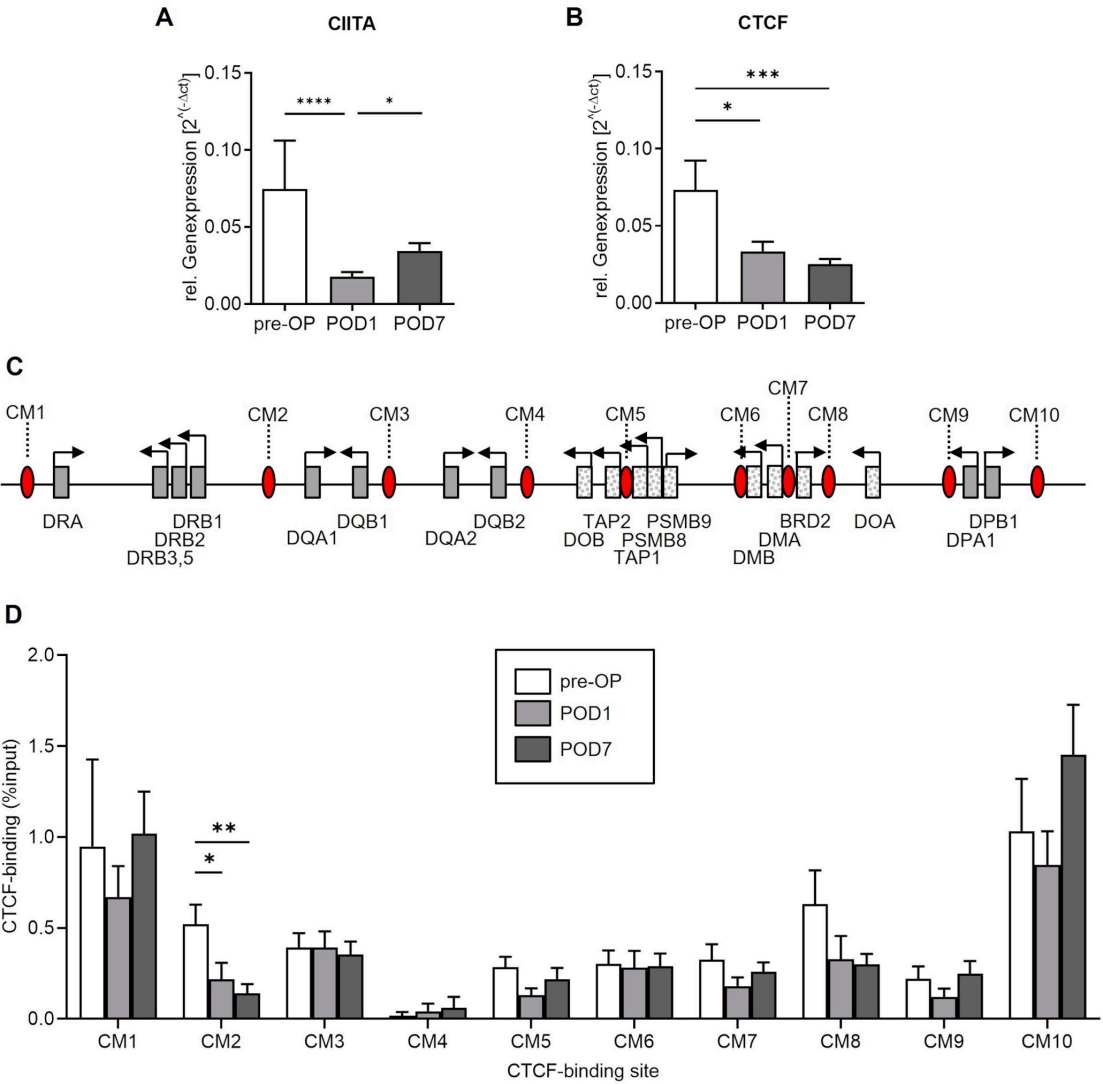
Expression of CIITA, CTCF and binding of CTCF within the MHC-II region. Expression of CIITA and the superordinate regulator protein CTCF and CTCF occupancy at specific binding sites within the MHC-II region were analyzed before surgery (`pre-OP´) as well as on postoperative days one (`POD1´) and seven (`POD7´). Relative gene expression of (A) CIITA and (B) CTCF (****p<0.0001, ***p<0.001, *p<0.05, Mann-Whitney U test, n = 10 patients, mean + SEM). (C) Schematic diagram of human chromosome 6p21.3 showing the relative positions of CTCF binding sites (CM1-CM10, red filled ovals) and MHC-II genes (boxes, classical HLA genes are shown as filled boxes). (D) Enrichment of CTCF at all known binding sites within the MHC-II region (**p<0.01, *p<0.05, Mann-Whitney U test, n = 10 patients, mean + SEM).

As shown in **Fig 4A and 4B**, surgery was associated with a decline in *CIITA* and *CTCF* expression (****p<0.0001 and *p<0.05, pre-OP vs POD1).

To investigate the effect of major abdominal surgery on the occupancy of CTCF at its binding sites inside the MHC-II (**Fig 4C**), we then conducted classical ChIP-experiments. Highest enrichment levels of CTCF were found at the two binding sites that flank the entire MHC-II region (CM1 and CM10). Compared to pre-operative baseline levels, patients displayed a marked reduction of CTCF occupancy at CM2, which is located in the intergenic region between *HLA-DRB1* and *HLA-DQA1*, on POD1 (*p<0.05, pre-OP vs POD1). In contrast, no differential CTCF binding occurred at the other analysed loci following surgery (**Fig 4D**).

Further experiments using primers against specific positive and negative control regions outside the investigated MHC subregion were performed to rule out a potential bias due to differential *CTCF* expression. There was no relevant CTCF occupancy within the negative control gene *MB*. In addition, neither *MB*, nor the positive control region H19ICR differed in CTCF enrichment at the observed time points (**S3 Fig**).

### Time-course of immune function: Surgery-induced loss of antigen presentation capacity does not fully recover to baseline levels within one week after surgery

Additional blood samples were taken at POD7 (**Fig 1**). While elevated plasma levels of pro-inflammatory IL-6 significantly dropped to preoperative levels, anti-inflammatory IL-10 concentrations remained low over time (**Fig 2A and 2B**). At POD7, ex vivo LPS stimulation induced a strong increase in both IL-6 and IL-10 levels (**Fig 2C and 2D**).

With a mean of 13,182 molecules per monocyte at POD7, surface HLA-DR density significantly increased compared to POD1 (*p<0.05, POD1 vs POD7) but did not reach pre-operative baseline levels (**p<0.01, pre-OP vs POD7, **Fig 3A**).

A partial transcriptional recovery of HLA-DR isotypes was observed (**Fig 3C and 3D**), while expression levels of both *HLA-DPA1* and *HLA-DPB1* remained decreased at POD7 (*p<0.05 and **p<0.01, pre-OP vs POD7; p>0.05, POD1 vs POD7, **Fig 3E and 3F**).

*CIITA* transcription recovered (p>0.05, pre-OP vs POD7; *p<0.05, POD1 vs POD7; **Fig 4A**), whereas *CTCF* expression remained reduced at POD7 (***p<0.001, pre-OP vs POD7; p>0.05, POD1 vs POD7 **Fig 4B**). Moreover, low CTCF binding at CM2 persisted at POD7 (**p<0.01, pre-OP vs POD7; p>0.05, POD1 vs POD7; **Fig 4C and 4D**).

## Discussion

The DNA-binding protein CTCF has been identified as superordinate genome-wide transcriptional regulator in various cell types, including those of the innate immune system. In functionally impaired CD14^+^-monocytes of patients suffering from abdominal sepsis, we previously found a differential and persisting binding of CTCF at specific target sites inside the MHC-II region with impact on HLA-gene expression [29,30]. Here, we investigated to which extent transcriptional MHC-II regulators, including CIITA and CTCF, as well as the expression of HLA-genes required for the process of antigen presentation are affected in non-immunocompromised patients undergoing elective major abdominal surgery. Compared to preoperative baseline levels, we found a surgery-induced decline in the transcription levels of *CIITA*, *CTCF* as well as the classical MHC-II genes *HLA-DRA*, *HLA-DRB1*, *HLA-DPA1* and *HLA-DPB1* that code for proteins expressed on monocyte surface. Furthermore, surgery was followed by a lower CTCF enrichment at an intergenic sequence within the HLA-DR subregion. This was previously shown to modulate MHC-II transcription via interaction with spatially related HLA-gene promoters [22,23]. To examine the time-course of the observed alterations in immune function, additional blood samples were taken seven days after surgery. Surgery-induced elevated plasma levels of pro-inflammatory IL-6 significantly dropped to preoperative levels, whereas anti-inflammatory IL-10 concentrations remained low over time. At POD7, ex vivo LPS stimulation induced a strong increase in both IL-6 and IL-10 levels. Daily measurements of HLA-DR surface expression via flow cytometry detected a slow and incomplete recovery of global immune competence during the study period, indicating a prolonged surgery-associated reduction of antigen-presenting capacity and immune competence. In line with the observed changes in HLA-DR receptor density, we found a partial transcriptional recovery of HLA-DR isotypes. In contrast to *HLA-DRA* and *HLA-DRB1*, expression levels of both *HLA-DPA1* and *HLA-DPB1* remained decreased at POD7. Regarding the investigated MHC-regulators, a recovery of *CIITA* transcription was detected, whereas in contrast, *CTCF* expression remained reduced at POD7. This was accompanied by persistent low CTCF-occupancy at the intergenic region CM2 within the MHC-II region.

Taken together, our findings support the hypothesis that after major abdominal surgery, CTCF is critically involved in MHC-II regulation of antigen presenting cells. The observation of *CTCF* expression and binding remaining altered beyond the typical life span of the investigated cells further indicates that CTCF contributes to a prolonged HLA-DR-suppressive immune phenotype and a higher vulnerability to infectious complications following surgical interventions.

It is well known that surgical injury induces the activation of immune cells and the release of various humoral mediators. This stress response aims to protect the body against pathogens and initiates the process of healing [1]. Among the involved cytokines, pro-inflammatory IL-6 levels, which were elevated on POD1 in the present collective, have been shown to correlate with the magnitude of surgical trauma [31–33]. Concerning anti-inflammatory IL-10, our patients displayed low plasma concentrations at all investigated time-points. Overall, our analysis including *ex vivo* stimulation experiments revealed a sustained perioperative reactivity of circulating immune cells to a secondary inflammatory stimulation.

However, in accordance with others, we detected a substantial surgery-induced drop of monocyte HLA-DR receptor density [8–12]. In our collective, this was followed by a slow and incomplete recovery during the observation period. This hints towards a prolonged impairment of key immune functions following major surgical injury, including the recognition and presentation of antigens on monocyte surface. Importantly, a recent study monitoring monocyte HLA-DR receptor density expression over time revealed that HLA-DR kinetics are associated with survival in patients with septic shock [34]. In line with own previous work in patients with postoperative sepsis [30], delayed or failing restoration of HLA-DR surface expression were found in patients with fatal outcomes [34]. These findings underline the need for a better understanding of the processes modulating MHC-II expression following surgical injury as well as during critical illness.

The present investigation of the MHC-II region and its superordinate regulators revealed several important findings: First, we found that major abdominal surgery did not globally affect MHC-II transcription but rather induced specific changes of selected HLA genes. Second, among the observed alterations, not all recovered in a timely consistent manner with some even persisting throughout the study period. In detail, in accordance with previous findings in septic patients with suppressed HLA-DR-expression [29,30], the decline in monocyte HLA-DR receptor density was associated with both reduced *CIITA* transcription as well as reduced mRNA levels of classical HLA-genes. However, instead of a general MHC-II downregulation, surgery induced a particular decrease in the expression of classical HLA-DR and -DP isotypes. Moreover, while the expression of *CIITA*, *HLA-DRA* and *HLA-DRB1* partially recovered, the detected transcriptional alterations of *HLA-DPA1* and *-DPB1* persisted. These findings corroborate the hypothesis of a dynamic and far more comprehensive transcriptional MHC-II control following major surgery that includes further players like the superordinate regulator CTCF [29,30].

Remarkably, in the human B lymphoblastoid cell line Raji, siRNA-mediated *CTCF* knockdown was found to impair HLA-gene expression [22]. The finding of CTCF presence being essential for MHC-II transcription is supported by the observations in the present study. Here, we were able to show that surgery-associated loss of *CTCF* expression was accompanied by reduced mRNA levels of classical HLA-genes.

Among the numerous CTCF targets spread across the human genome, ten sites were confirmed within the human MHC-II region, located between genes coding for different HLA-isotypes and non-HLA expressing genes [22]. Of note, we found that surgery exclusively decreased CTCF occupancy at a highly acetylated X box-like chromatin insulator element first described by Majumder and Boss ([23]; termed CM2 in the present study), which is located at the boundary between the HLA-DR- and HLA-DQ-subregions. In Raji cells, the CTCF binding sites identified within the MHC-II region including CM2 were found to interact with each other as well as with spatially related HLA promoter regions [22,28,35]. Further cell-culture experiments revealed that *CTCF* knockdown induces a loss of the described three-dimensional interactions between CM2 and other CTCF-sites with subsequent impact on HLA-gene expression [22]. The particular relevance of the ~47kB spanning intergenic region between *HLA-DRB1* and *HLA-DQA1* in MHC-II regulation is underlined by the presence of two recently discovered super-enhancers (SE), one encompassing CM2 [36]. Using CRISP/Cas9, an interaction between the two SE, as well as HLA-gene promoters was detected. Besides, the DR/DQ-SE was found to interact with other CTCF targets within MHC-II [36]. Together with the present findings as well as own previous observations in patients with sepsis [29,30], this supports the hypothesis of a complex regulatory model, where CTCF may regulate gene expression by modulating the topological architecture of the entire MHC-II region. Intriguingly, our study reveals several differences concerning *CTCF* expression and its binding patterns within the MHC-II in non-immunocompromised patients undergoing elective major abdominal surgery compared to critically ill patients suffering from postoperative abdominal sepsis [29,30].

As another important finding, we could show that diminished binding of CTCF within the MHC-II region as well as transcriptional changes of the investigated HLA-DP genes remained observable even seven days after surgery. Since in healthy humans the lifespan of circulating CD14^+^-monocytes commonly ranges between 1–4 days [37], the monocytes isolated at POD7 in the present study most likely descended from later generations then those isolated before surgery and at POD1. We therefore speculate that major abdominal surgery induces sustained epigenetic alterations with prolonged impact on MHC-II transcription, possibly involving differential CTCF binding patterns in hematopoietic precursor cells.

There are some limitations to this study. First, we exclusively investigated monocytes in patients undergoing major abdominal surgery. Consequently, the results are not generalizable to (i) other cell types or (ii) other medical interventions with potential impact on immune function, i.e. major orthopedic or cardiac surgery. Additional studies are needed to elucidate which component(s) of the surgical procedure including anesthesia cause the observed alterations. Second, we excluded patients taking immune-suppressive medication, yet included patients suffering from cancer with some having received chemotherapy before surgery–conditions known to modulate the immune system. Thus, the findings of the present study cannot be generalized to patients with differing immune states. Furthermore, since the experimental workflow used in this work necessitated immediate processing of the blood samples, the study was designed as a single center observation to reduce the risk of technical bias. Finally, as a pilot study, a comparably small number of patients were investigated. The results of this study therefore need further validation using larger cohorts.

In summary, our study provides additional insights into the regulatory system controlling transcription of MHC-II components. Our findings indicate that beside CIITA, the superordinate regulator CTCF is critically involved in MHC-II regulation of circulating human monocytes following major abdominal surgery. Instead of global effects on MHC-II transcription, we identified specific changes of selected HLA genes. The observed alterations did not recover in a timely consistent manner with some even persisting throughout the study period, contributing to a HLA-DR suppressive phenotype that might increase the patients`vulnerability following major surgical interventions.

## Methods

### Ethics and patients

This study was conducted between 07/2018 and 08/2019 in accordance with the principles expressed in the Declaration of Helsinki after approval of the ethics committee of the medical faculty of Heidelberg University (Alte Glockengießerei 11/1, D-69115 Heidelberg, Germany; approval number S-135/2016) and after registration in the German Clinical Trial Register (Trial number: DRKS00011667). During and after the study implementation, authors had access to information that could identify individual participants. Data protection protocols for this study adhere to current applicable regulations of the Federal and State Data Protection Act of Baden-Württemberg and the `Datenschutzgrundverordnung´; including protection against unauthorized access and data loss. Participants were at least 18 years old and provided written informed consent on forms approved by the local ethics committee. All patients underwent elective major abdominal surgery at the Surgery Center of Heidelberg University Hospital. Exclusion criteria were participation in an interventional study, non-elective surgery, autoimmune diseases and / or immune-suppressive medication, pre-existing renal failure, infectious diseases (i.e. HIV, viral hepatitis) or laboratory signs of infection.

### Data collection, standard laboratory parameters and experimental workflow

Study-related data, including demographics and pre-existing comorbidities (based on the Charlson comorbidity index [38]) were extracted from electronic and paper-based medical records. According to in-house procedures, standard laboratory parameters were measured in the routine hospital laboratory. Blood samples were taken directly before induction of anesthesia (`pre-OP´) as well as on postoperative days one (`POD1´) and seven (`POD7´) and were immediately processed in the experimental workflow. In addition, daily measurements of monocyte HLA-DR surface expression were performed until POD7 (**Fig 1**). Part of the experimental data of POD7 served as control values in previously published work to investigate an independent scientific research question [30].

### Monocyte surface expression of HLA-DR

Flow cytometry using a FACSVerseTM flow cytometer (BD Bioscience, Heidelberg, Germany) was conducted to quantify the expression of HLA-DR on CD14^+^-monocyte surface as previously described [30]. In particular, incubation of 50 μl ETDA-anti-coagulated whole blood with 20 μl anti-HLA-DR antibody (Quantibrite anti-HLA-DR/Monocyte antibody, BD Bioscience, Heidelberg, Germany) was followed by erythrocyte lysis (FACS Lysing solution, BD Bioscience, Heidelberg, Germany) according to manufacturer`s instructions. After identifying monocytes based on CD14 expression, surface expression of HLA-DR was measured as median fluorescence intensity (MFI). Conversion of determined MFI-values using a 4-point calibration curve (Quantibrite PE Beads, BD Bioscience, Heidelberg, Germany) allowed quantification of HLA-DR molecules on cell surface (`counts per monocyte´).

### Monocyte separation

Monocyte separation and quality control was performed as previously described [30]. In brief, peripheral blood mononuclear cells were separated from a 30 ml Lithium-heparin-anti-coagulated blood sample via Ficoll-based density gradient centrifugation. CD14^+^-monocytes were isolated using magnetic cell sorting (autoMACS, Miltenyi Biotec, Bergisch Gladbach, Germany) with specific CD14-MicroBeads (Miltenyi Biotec, Bergisch Gladbach, Germany). A small quality control sample was incubated with anti-human-CD14-FITC antibody (BioLegend, San Diego, USA) and used for flow cytometry (FACSVerseTM flow cytometer, BD Bioscience, Heidelberg, Germany) to ensure purity of the isolated cells.

### Gene expression analysis

Gene expression analysis was performed following RNA extraction from isolated CD14^+^-monocytes using a column-based method (RNeasy Plus Mini Kit, Qiagen, Hilden, Germany) according to manufacturer`s instructions. Sufficient quality and concentration of the extracted RNA was confirmed via spectrophotometry (Nanodrop, Thermo Fisher Scientific, Waltham, USA). All RNA samples used for subsequent reverse transcription showed 260/280 as well as 260/230 ratios above 1.8. From each sample, 1 μg RNA was transcribed into cDNA (Quantitect Reverse Transcription Kit, Qiagen, Hilden, Germany). Quantitative PCR experiments (StepOnePlusTM PCR-cycler, Applied Biosystems, Foster City, USA) were conducted with commercially available TaqMan assays (Applied Biosystems, Foster City, USA) and reagents (TaqMan Gene Expression Master Mix, Applied Biosystems, Foster City, USA, **S2 Table**).

Reactions were done in triplicate. For each gene of interest, mean Ct values were calculated and subtracted from the mean of the two endogenous control genes *Actin Beta* (*ACTB*) and *Hypoxanthine Phosphoribosyltransferase 1* (*HPRT1*) for ΔCt analysis. Relative gene expression was then calculated by 2^ΔCt^.

### Chromatin immunoprecipitation and quantitative PCR analysis

Chromatin immunoprecipitation (ChIP) was performed as previously published [30]. DNA-protein links of isolated CD14^+^-monocytes were fixed by incubation with formaldehyde (final concentration 1%) for 10 minutes at 18°C. Addition of glycine at a final concentration of 0.125 M stopped the cross-link reaction. Cell lysis (10^6^ cells per 200 μl lysis buffer) was followed by DNA-shearing under constant cooling (Bioruptor Pico®, Diagenode, Liège, Belgium; fragment size 150–200 base pairs). Subsequent steps including immunoprecipitation, reverse cross-linking and DNA purification were conducted using an automated system (IP-Star® Compact), associated reagent kits (Auto iDeal ChIP-seq Kit for Transcription Factors) as well as specific ChIP antibodies against CTCF (all obtained from Diagenode, Liège, Belgium; **S2 Table**) according to manufacturer`s instructions. QubitTM assay (Qubit Fluorometric Quantitation, Thermo Fisher Scientific, Waltham, USA) served for quantification of the obtained ChIP-DNA (diluted in pure H_2_O). Quantitative PCR experiments were done on a StepOnePlusTM PCR-cycler (Applied Biosystems, Foster City, USA) using specific primer sets for CTCF binding sites within the MHC-II region termed CM 1–10 (as previously identified by Majumder and Boss [22]). Primers against specific positive and negative control regions outside the MHC-II region (positive control: H19/IGF2 Imprinting Control Region (H19ICR), located on chromosome 11; negative control: *Myoglobin* (*MB*), located on chromosome 22) served to address assay background and to rule out a potential bias due to differential CTCF expression. Primer sequences are listed in Table C in **S2 Table**.

### Ex vivo whole blood stimulation

For functional assessment of circulating immune cells, whole blood (collected in Lithium-heparin-anti-coagulated tubes) was diluted 1:1 with RPMI1640 containing proprietary GlutMAX™ (Thermo Fisher Scientific, Waltham, MA, USA) and 5% fetal bovine serum (Ultra-low endotoxin; Cell Concepts GmbH, Umkirch, Germany) followed by incubation with ultrapure lipopolysaccharide (LPS, Invivogen, San Diego, CA, USA) in a final concentration of 100 ng/ml medium. After 24 hours, samples were centrifuged (5,000 rpm, 5 minutes) and supernatants were used for subsequent enzyme-linked immunosorbent assay (ELISA).

### Enzyme-linked immunosorbent assay

Concentrations of pro- and anti-inflammatory cytokines (IL-6, IL-10) were determined via ELISA using both plasma derived from centrifuged whole blood as well as supernatants from ex vivo stimulated samples according to manufacturer`s instructions (R&D Technologies, North Kingstown, USA).

### Statistical analysis and data visualization

Statistical analysis and data visualization were conducted using GraphPad Prism (Version 6.0f, GraphPad Software, La Jolla, USA). Raw data are provided in **S3 Table**. Fisher’s exact test and Mann-Whitney U test were used to compare categorical and continuous data, respectively. Results are visualized as bars and expressed as mean and standard error of the mean (SEM). P values <0.05 were considered significant and indicated with ‘*’ (p<0.05, >0.01), ‘**’ (p<0.01), ‘***’ (p<0.001) or ‘****’ (p<0.0001).

## Supporting information

S1 Fig Individual delta of cytokine secretion after LPS-stimulation compared to unstimulated control. Cytokine levels in plasma and supernatants from ex-vivo stimulated samples before surgery (`pre-OP´) as well as on postoperative day one (`POD1´). Individual delta in (A) IL-6 secretion and (B) IL-10 secretion compared to unstimulated control in supernatants after ex-vivo stimulation with bacterial lipopolysaccharide (LPS) is shown (p>0.05, Mann-Whitney U test, n = 10 patients, mean + SEM).
(TIF)

S2 Fig Expression of HLA-DR and HLA-DP subunits. Samples were analyzed before surgery (`pre-OP´) as well as on postoperative days one (`POD1´) and seven (`POD7´). RNA from isolated human CD14^+^-monocytes was used for reverse transcription and subsequent qPCR experiments using TaqMan Assay against classical HLA-DR and -DQ subtypes HLA-DRB3 (A), HLA-DRB5 (B), HLA-DQA1 (C), HLA-DQB1 (D), HLA-DQA2 (E) and HLA-DQB2 (F; p>0.05, Mann-Whitney U test; n = 10 patients, mean + SEM).
(TIF)

S3 Fig CTCF binding at specific control regions outside the MHC-II region. Chromatin from isolated human CD14^+^-monocytes (pre-OP, POD1 and POD7) was immunoprecipitated with anti-CTCF antibody for subsequent qPCR using primer pairs selected genome regions serving as positive and negative controls. H19-ICR (located on chromosome 11) served as positive control for CTCF-binding. *MB* (located on chromosome 22) served as negative control for CTCF binding (p>0.05, Mann-Whitney U test, n = 10 patients, mean + SEM).
(TIF)

S1 Table Socio-demographic and clinical characteristics of the study population. (XLSX)

S2 Table Assays, antibodies and primers. Table A: TaqMan^TM^ assays. Table B: ChIP antibodies. Table C: ChIP-qPCR primers.
(XLSX)

S3 Table Raw data. (XLSX)
