# Supplementary figures and images for: Abdominal surgery induces long-lasting changes in expression and binding of CTCF with impact on Major Histocompatibility Complex II transcription in circulating human monocytes
Source: PLoS One. 2023 Oct 25;18(10):e0293347. doi: 10.1371/journal.pone.0293347 (PMC10599505; doi:10.1371/journal.pone.0293347)

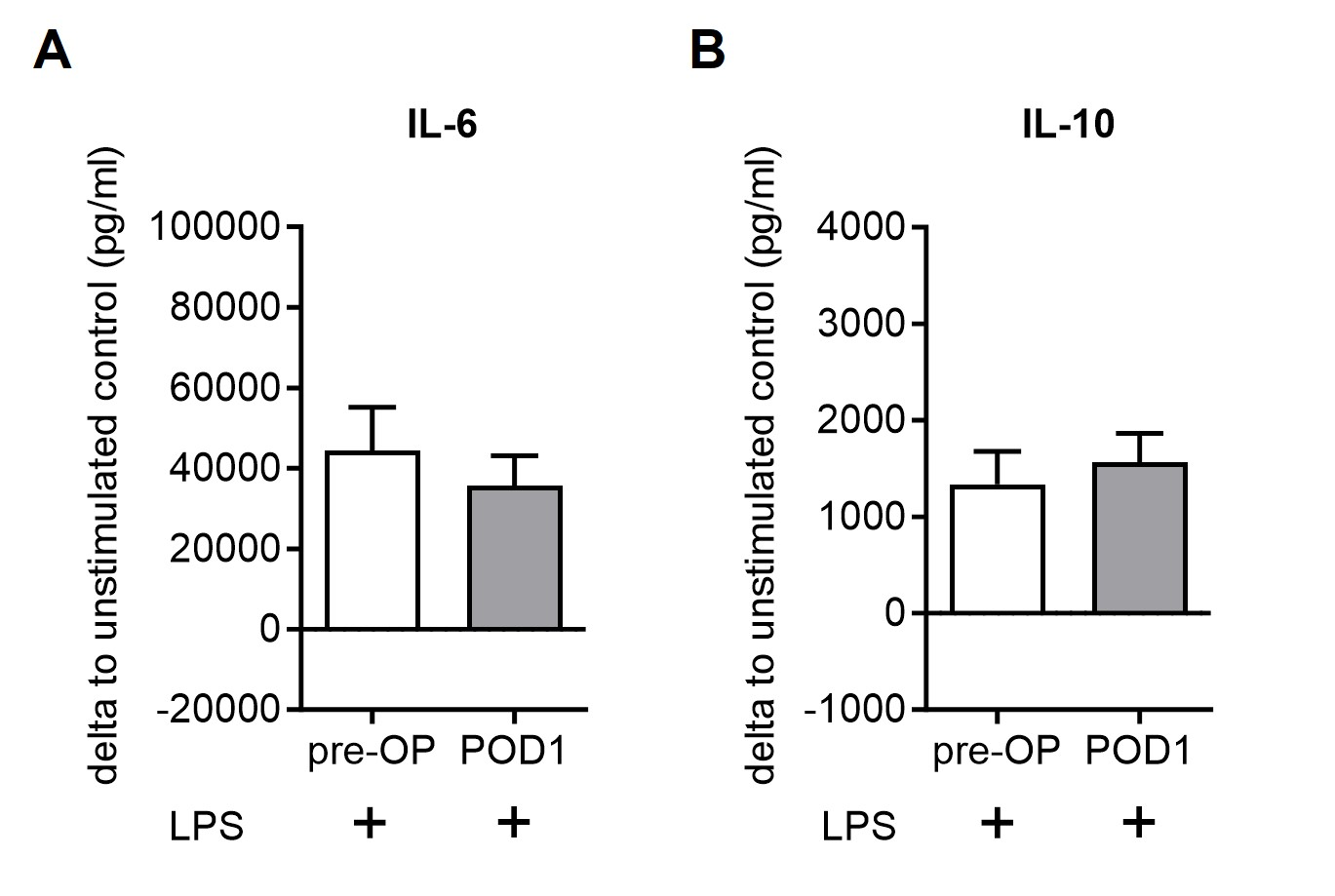

Supplement: S1 Fig — Cytokine levels in plasma and supernatants from ex-vivo stimulated samples before surgery (`pre-OP´) as well as on postoperative day one (`POD1´). Individual delta in (A) IL-6 secretion and (B) IL-10 secretion compared to unstimulated control in supernatants after ex-vivo stimulation with bacterial lipopolysaccharide (LPS) is shown (p>0.05, Mann-Whitney U test, n = 10 patients, mean + SEM). (TIF) [file pone.0293347.s001.tif]

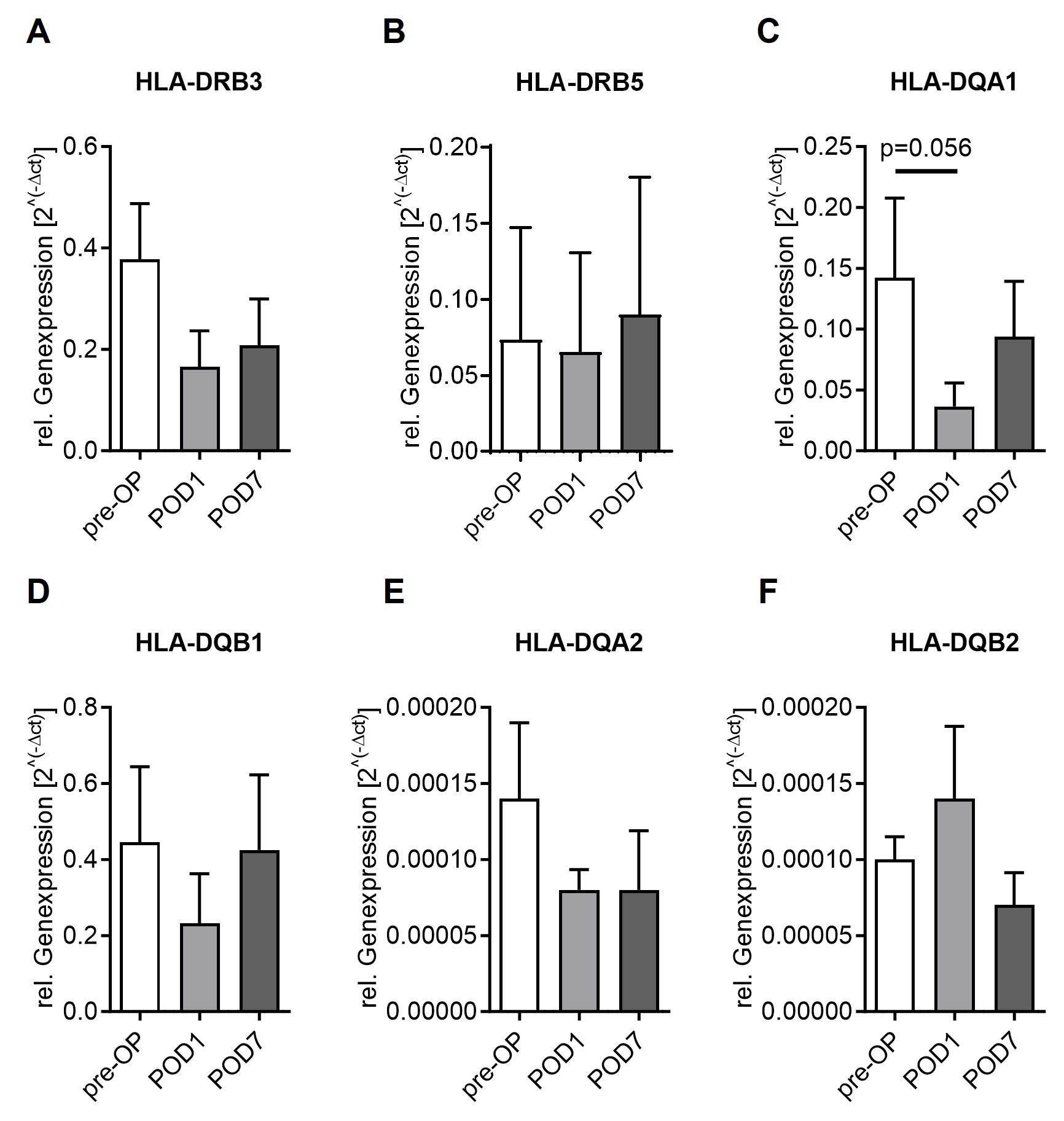

Supplement: S2 Fig — Samples were analyzed before surgery (`pre-OP´) as well as on postoperative days one (`POD1´) and seven (`POD7´). RNA from isolated human CD14+-monocytes was used for reverse transcription and subsequent qPCR experiments using TaqMan Assay against classical HLA-DR and -DQ subtypes HLA-DRB3 (A), HLA-DRB5 (B), HLA-DQA1 (C), HLA-DQB1 (D), HLA-DQA2 (E) and HLA-DQB2 (F; p>0.05, Mann-Whitney U test; n = 10 patients, mean + SEM). (TIF) [file pone.0293347.s002.tif]

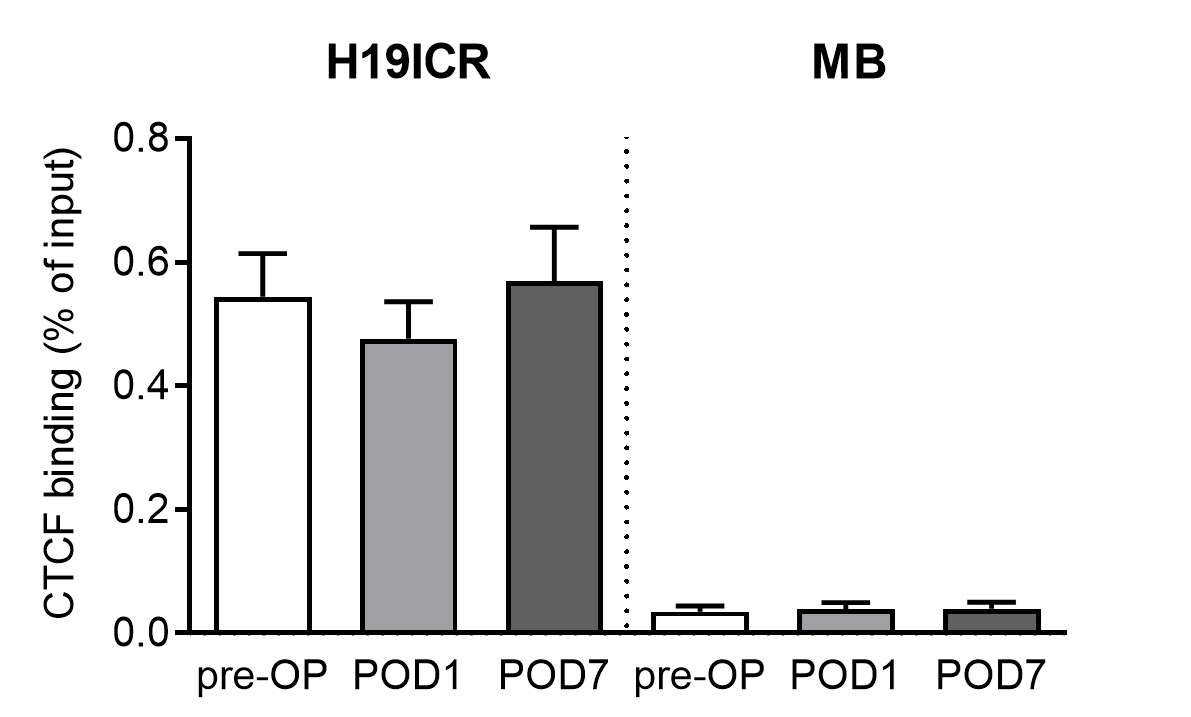

Supplement: S3 Fig — Chromatin from isolated human CD14+-monocytes (pre-OP, POD1 and POD7) was immunoprecipitated with anti-CTCF antibody for subsequent qPCR using primer pairs selected genome regions serving as positive and negative controls. H19-ICR (located on chromosome 11) served as positive control for CTCF-binding. MB (located on chromosome 22) served as negative control for CTCF binding (p>0.05, Mann-Whitney U test, n = 10 patients, mean + SEM). (TIF) [file pone.0293347.s003.tif]
